# Repurposing Alzheimer’s disease medications—systemic cholinesterase inhibitors—for the treatment of dry eye syndrome, glaucoma, and age-related macular degeneration

**DOI:** 10.1177/25158414251341022

**Published:** 2025-05-16

**Authors:** Shilpa Rajagopal, Pranati Ahuja, Valerie Quach, Cody Luong, Mukaila A. Raji

**Affiliations:** John Sealy School of Medicine, UTMB, 301 University Boulevard, Galveston, TX 77555, USA; Department of Ophthalmology, UTMB, Galveston, TX, USA; John Sealy School of Medicine, UTMB, Galveston, TX, USA; John Sealy School of Medicine, UTMB, Galveston, TX, USA; Division of Geriatric, Medicine Department of Internal Medicine, UTMB, Galveston, TX, USA

**Keywords:** age-related, cholinesterase inhibitor, dry eye syndrome, glaucoma, macular degeneration

## Introduction

Optimal vision is key to promoting physical and mental well-being and a good quality of life among older adults. Vision loss and low vision can substantially impact the completion of activities of daily living (ADLs) and participation in social and leisure pursuits. Eye conditions such as dry eye syndrome (DES), glaucoma, and age-related macular degeneration (ARMD) frequently present in older age and are common contributors to poor quality of life. In particular, glaucoma and ARMD are progressive disease states, contributing to vision loss and disability, outcomes that are exacerbated by disparate access to healthcare resources.^1^ These diseases commonly co-occur, in part due to increasing prevalence with age, and data suggest that there is a link between DES and glaucoma, likely attributed to adverse medication effects of glaucoma drugs that can increase irritation to and dryness of the eye.^2^ Moreover, eye diseases such as cataracts, glaucoma, and ARMD have been found to exist alongside dementia and neurodegenerative states.^3^ Available treatments can differ for each of these eye conditions, ranging from lifestyle modifications and artificial tear eye drops for DES; to alpha-adrenergic agonists, beta-blockers, and miotics for glaucoma, and antioxidant supplementation for dry ARMD, among others.^4,5^ In other words, an older adult with two or more of these conditions can potentially be taking five or more different ophthalmic medications, contributing to ophthalmic polypharmacy. These eye medications are in addition to other medications that older adults may be prescribed for multiple other chronic conditions (e.g. hypertension, diabetes, and Alzheimer’s disease) that are common in this patient population. The polypharmacy and multimorbidity contexts increase the risk of adverse drug-drug and drug-disease interactions as well as medication nonadherence. A potential avenue to reduce ophthalmic polypharmacy is the use of one medication that can be harnessed or “repurposed” to address multiple eye conditions and symptoms.^6^ This paper explores the potential of adopting a single drug class—the acetylcholinesterase inhibitors FDA-approved for Alzheimer’s dementia—as a potential one-stop medication to help manage DES, glaucoma, and ARMD. Such an approach has the potential to reduce polypharmacy, adverse drug events, costs, and medication nonadherence. We present evidence from both basic and clinical literature on the mechanisms of action of acetylcholinesterase inhibitors associated with beneficial effects on these eye conditions.

## Mechanism of action of acetylcholinesterase inhibitors: Implications for management of common eye diseases

**Figure 1.**
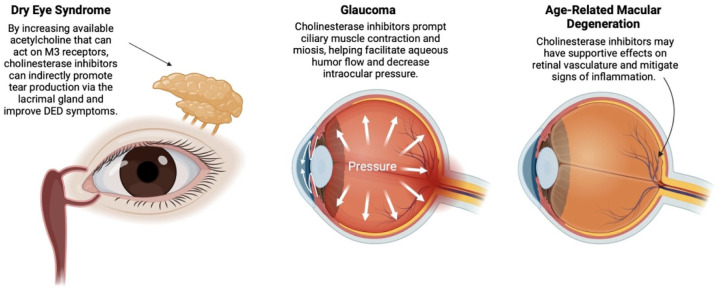
Proposed mechanisms by which acetylcholinesterase inhibitors (AChEI) affect the pathophysiology of dry eye syndrome (DES), glaucoma, and age-related macular degeneration (ARMD). Source: Images adapted from and created in BioRender.com. AChEI, acetylcholinesterase inhibitors; ARMD, age-related macular degeneration.

Acetylcholinesterase inhibitors (AChEI) function by blocking acetylcholinesterase, an enzyme that breaks down acetylcholine at the neuromuscular junction.^7^ As a result, AChEI increase acetylcholine levels, which act on muscarinic receptors and contribute to lacrimation, miosis, and subsequent decreases in intraocular pressure. Prior research has demonstrated associations between acetylcholine and ophthalmologic function (see Figure 1).^8^ Laspas et al. (2019) showed that loss of the M1 muscarinic receptor in 15-month-old mice was linked to fewer retinal ganglion cell neurons and optic nerve axons.^9^ Currently, cholinesterase inhibitors are used for the management of several diseases, including ocular myasthenia gravis, often treated with pyridostigmine and Alzheimer’s dementia. This is particularly noteworthy given the focus on older adult patients, who are more likely to be diagnosed with dementia alongside other eye-related pathologies and may already be taking cholinesterase inhibitors, thus reducing the polypharmacy burden.^10,11^ Major cholinesterase inhibitors approved for Alzheimer’s dementia management include galantamine, donepezil, and rivastigmine. Galantamine and donepezil, in particular, have been found to lessen optic nerve axonal degeneration and improve brightness discrimination following optic nerve injury, respectively.^12,13^ Side effects of the drugs are associated with cholinergic toxicity, which can present as diarrhea, urinary incontinence, bradycardia, and excess secretions.

## Role of AChEI for eye diseases

There are few human subject and animal model trials examining the efficacy of cholinesterase inhibitors for DES, glaucoma, and ARMD. Yet, the current evidence in support of this intervention is promising. Below we describe evidence supporting AChEI treatment potential for each of the eye conditions.

## Repurposing AChEI for dry eye syndrome

A study examining mice with a knockout M3 muscarinic receptor compared to wild-type mice found decreased lacrimation, increased inflammatory cytokine expression, and reduced corneal epithelial microvilli length among 15-month-old mice without the M3 receptor.^14^ The latter findings are suggestive of the potential to repurpose AChEI for DES, underscoring the role of the acetylcholine pathway in the pathophysiology of DES. This is further supported by the fact that increased tearing, which can potentially alleviate dry eye symptoms, is a known side effect of AChEI medications due to parasympathetic activation pathways.^14^ While the use of an M3 receptor agonist has demonstrated improvement in dry eye conditions, data specific to cholinesterase inhibition and DES is still limited.^8^

## Repurposing AChEI for glaucoma

Cholinesterase inhibitors have also been associated with positive outcomes for both glaucoma and ARMD. Glaucoma is a common eye condition characterized by increased intraocular pressure, which can lead to permanent vision loss due to the destruction of retinal ganglion cells.^15^ Historically, AChEI were used to manage open-angle glaucoma by inducing ciliary muscle contraction, thereby promoting aqueous humor drainage and lowering intraocular pressure.^15^ Data suggest that glaucoma-induced vision loss stems not only from retinal ganglion cell damage but also from microvascular injury.^16^ One such study demonstrated that the vasodilatory effects of the cholinesterase inhibitor galantamine can also help preserve vision by maintaining microvasculature integrity in the eye.^16^ Of note, older adults may tend to experience senile miosis due to age-associated atrophy, resulting in decreased night vision capabilities. In the setting of this preexisting miosis, the use of a cholinesterase inhibitor can exacerbate miotic conditions and contribute to further vision reduction, especially in patients with narrow-angle glaucoma. For such individuals, examination of the full clinical picture and engagement in shared decision-making is critical; some patients with dementia symptoms affecting ADL and instrumental ADL completion may find greater therapeutic and logistical benefits from using a single cholinesterase inhibitor for dual purposes despite the associated side effects. In these instances, close monitoring of visual changes and coordination of care between geriatricians and ophthalmologists can be especially valuable.

## Repurposing AChEI for ARMD

With regards to ARMD, contributing factors to the development of the disease include oxidative damage, chronic inflammation, abnormalities in lipid metabolism, and structural changes.^17^ While more research is needed to establish definitive causal relationships, a recent study published in *JAMA Ophthalmology* (2024) indicates that AChEI may reduce the risk of developing ARMD in veterans with Alzheimer’s disease.^18^ One proposed mechanism discussing the benefits of cholinesterase inhibition in this condition is the suppression of inflammation, which could reduce cell death, choroidal neovascularization, and geographic atrophy.^18,19^ Another study highlighted the role of acetylcholine in retinal inflammation through NF-kB signaling.^20^ Thus, the use of common cholinesterase inhibitors among patients with identified neurocognitive changes who would already benefit from such medication may also offer a cost-effective approach to mitigate inflammation in the eye and potentially lower the risk of coexisting conditions, such as ARMD.

## Conclusion and clinical implications

The high prevalence of eye diseases such as DES, glaucoma, and ARMD among older adults highlights the importance of assessing current FDA-approved medications for their potential to be repurposed as a one-stop treatment for multiple eye conditions and symptoms. Such repurposing can reduce polypharmacy and drug toxicity and increase drug adherence in geriatric patients who frequently have multiple, chronic eye and non-eye conditions that can co-occur. The preliminary evidence presented above supports the potential benefits of AChEI for older patients with DES, glaucoma, and ARMD. While there are challenges associated with the conduct of clinical trials involving individuals with multimorbidity for these eye conditions in the setting of neurocognitive impairment, larger randomized controlled trials through interdisciplinary collaborations are needed to better understand the therapeutic effects of cholinesterase inhibitor dementia drugs on age-associated ophthalmologic diseases.

## Declarations

**Ethics approval and consent to participate:** Institutional Review Board approval was not applicable to this editorial. *Consent to participate:* Not applicable.

**Consent for publication:** Not applicable.

**Availability of data and materials:** Data sharing not applicable to this article as no datasets were generated or analyzed during the writing of this editorial.
